# Regressing Eruptive Disseminated Spitz Nevi: A Case Report

**DOI:** 10.7759/cureus.72707

**Published:** 2024-10-30

**Authors:** Lingling Yang, Yong Zhang

**Affiliations:** 1 Dermatology, The Third People's Hospital of Hangzhou, Hangzhou, CHN

**Keywords:** eruptive disseminated spitz nevi, gene mutation, long-term follow-up, regression, spitz nevus

## Abstract

Eruptive disseminated Spitz nevi (EDSNs) are clinically rare, but no malignant change has been reported. Most EDSNs remain stable, which affects their appearance and causes great psychological pressure on patients. We report the case of an 18-year-old man who suddenly developed a large number of pigmented nevi within 20 days. After a two-year follow-up, most of the nevi spontaneously resolved without any treatment.

## Introduction

Spitz nevi can be classified into solitary, agminated, and disseminated forms. Eruptive disseminated Spitz nevi (EDSNs) are less common, and only a few cases have been reported. EDSNs are usually characterized as a large number of pink or black nevi on the trunk and proximal limbs in a short period of time and remain stable for a long time, causing huge psychological pressure on patients. The precise pathogenesis of EDSNs is unknown, and no specific genetic mutation has been identified. The etiology may be related to human immunodeficiency virus (HIV) infection, adolescence, pregnancy, Addison's disease, trauma, or drugs, and some cases may be associated with atopic dermatitis [1-4]. At present, effective treatment hasn't been found, and reports of spontaneous resolution are rare.

## Case presentation

**Figure 1:**
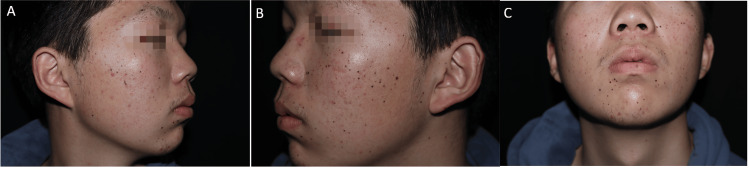
Clinical photographs A, B, and C: On dermatological examination, there were multiple irregular 1-3 mm brown and black macules on the face (date: 2022.01.26).

**Figure 2:**
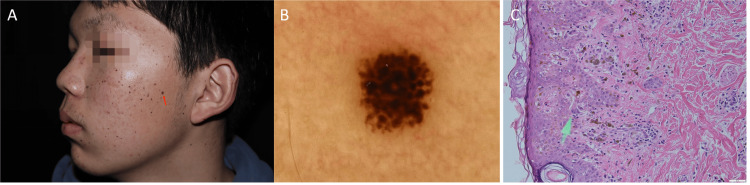
Dermoscopy and histological examination A: We selected a larger pigmented nevus (indicated by the red arrow) on the left side of the patient's face for skin dermoscopy and histological examination. B: Dermoscopy showing the starburst mode. C: Histological examination of the biopsy specimen from the face revealed hyperplasia of epithelial nevi cells at the basal layer of the epidermis (green arrow), but no cellular abnormalities (H&E ×200). H&E: hematoxylin and eosin

**Figure 3:**
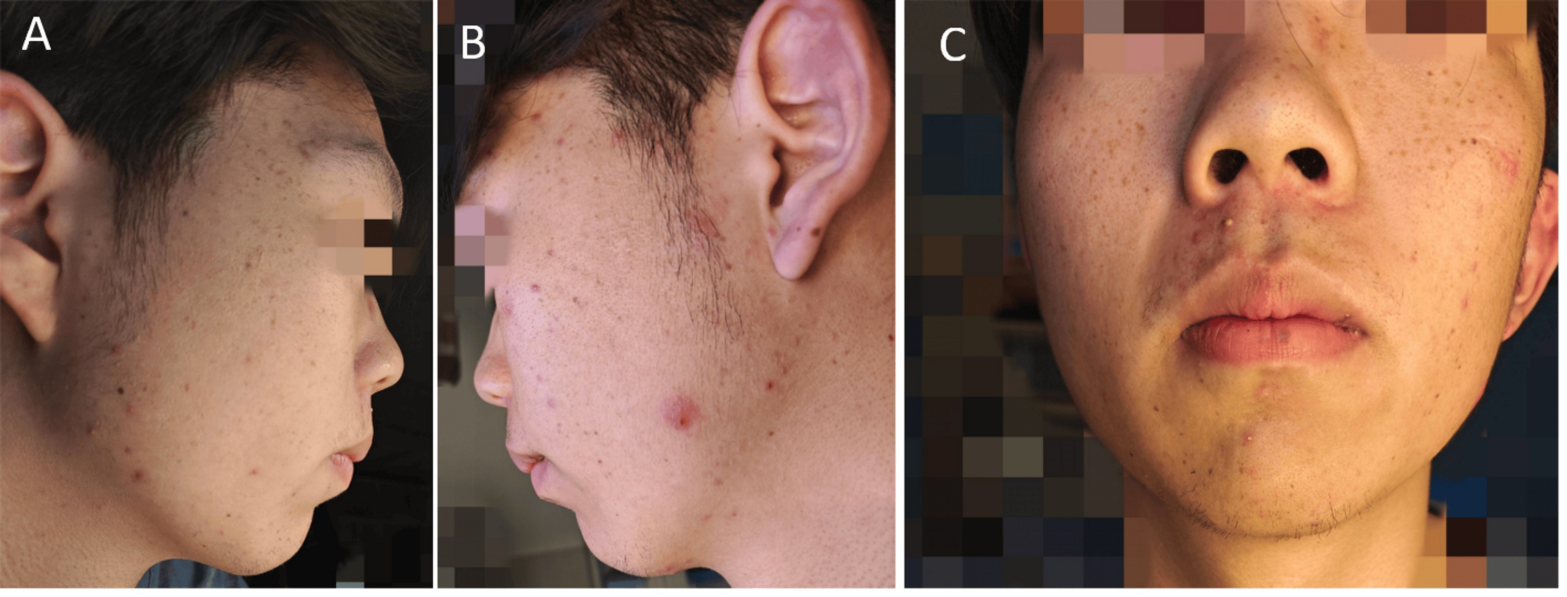
Clinical photographs A, B, and C: After two years of follow-up, most nevi faded spontaneously without any treatment (date: 2024.05.08).

An 18-year-old man presented to our outpatient clinic with numerous nevi on his face, neck, and trunk. Most lesions erupted abruptly in 20 days without any apparent cause and then kept stationary ever since. On dermatological examination, there were multiple irregular 1-3 mm brown and black macules on the face, neck, and trunk (Figure 1). The results of a comprehensive examination including gastrointestinal endoscopy and BRAF testing were negative. However, the KIT mutation was found in exon 17 (p.N822Y). Dermoscopy showed starburst mode (Figure 2). Histological examination of the biopsy revealed hyperplasia of epithelial nevi cells in the epidermis, but no cellular abnormalities were observed (Figure 2), which supported the diagnosis of Spitz nevi. Given the clinical manifestation and the results of the examination, the diagnosis of EDSN was certainly considered. After two years of follow-up, most nevi faded spontaneously without any treatment (Figure 3).

## Discussion

EDSNs are not common in clinical practice and occur more frequently in individuals between the ages of 10 and 20. They are most commonly found on the trunk and proximal limbs [5].

The pathogenesis of EDSNs is not yet clear. One proposed mechanism is that EDSN is caused by immunosuppression. In addition, some other studies have suggested that EDSN may be associated with mutations in various genes, including BRAF, ALK, ROS1, NTRK1, NTRK3, MERTK, MET, or RET [6,7]. However, the specific genetic mutations have not been found [8].

It is not feasible to remove all of the eruptive moles because the number of lesions can range from tens to hundreds. Most EDSNs were stable, with no reports of malignant transformation. Therefore, it is recommended to follow up patients regularly and remove the suspected lesions for malignancy in time, rather than advocating the use of non-surgical methods, including laser, etc. Several cases of spontaneous regression of EDSN have been reported previously [9-11]. In our patient, most lesions spontaneously regressed after two years of onset, indicating that spontaneous regression of EDSN is possible and long-term follow-up is a safe management method.

## Conclusions

We report an EDSN. Although no cases of malignant transformation have been reported, EDSN still causes great physical and psychological stress in patients. Only four cases of spontaneous regression of EDSN have been reported previously. In our case, most nevi regressed within two years without any treatment, demonstrating that close observation is the best management and complete spontaneous regression is possible. However, further case and genetic studies are necessary to understand the pathogenesis and prognosis of EDSN.
